# Tumor response evaluation in patients with malignant melanoma undergoing immune checkpoint inhibitor therapy and prognosis prediction using ^18^F-FDG PET/CT: multicenter study for comparison of EORTC, PERCIST, and imPERCIST

**DOI:** 10.1007/s11604-021-01174-w

**Published:** 2021-07-21

**Authors:** Kazuhiro Kitajima, Tadashi Watabe, Masatoyo Nakajo, Mana Ishibashi, Hiromitsu Daisaki, Fumihiko Soeda, Atsushi Tanemura, Takuro Kanekura, Naoya Yamazaki, Kimiteru Ito

**Affiliations:** 1 Department of Radiology, Hyogo College of Medicine, 1-1 Mukogawa-cho, Nishinomiya, Hyogo 663-8501 Japan; 2 Department of Nuclear Medicine and Tracer Kinetics, Graduate School of Medicine, Osaka University, Suita, 565-0871 Japan; 3 Department of Radiology, Graduate School of Medical and Dental Sciences, Kagoshima University, 8-35-1, Sakuragaoka, Kagoshima, 890-8544 Japan; 4 Division of Radiology, Department of Pathophysiological and Therapeutic Sciences, Tottori University, 86 Nishi-cho, Yonago, Tottori 683-8503 Japan; 5 Graduate School of Radiological Technology, Gunma Prefectural College of Health Science, 323-1 Kamioki machi, Maebashi, Gunma 371-0052 Japan; 6 Department of Dermatology, Graduate School of Medicine, Osaka University, Suita, 565-0871 Japan; 7 Department of Dermatology, Graduate School of Medical and Dental Sciences, Kagoshima University, Kagoshima, 890-8544 Japan; 8 Department of Dermatologic Oncology, National Cancer Center Hospital, 5-1-1 Tsukiji, Chuo-ku, Tokyo, 104-0045 Japan; 9 Department of Diagnostic Radiology, National Cancer Center Hospital, 5-1-1 Tsukiji, Chuo-ku, Tokyo, 104-0045 Japan

**Keywords:** Malignant melanoma, ICI (immune checkpoint inhibitor), FDG (fluorodeoxyglucose), PET/CT (positron emission tomography/computed tomography), EORTC (European Organization for Research and Treatment of Cancer), PERCIST (Positron Emission Tomography Response Criteria in Solid Tumors)

## Abstract

**Objective:**

In malignant melanoma patients treated with immune checkpoint inhibitor (ICI) therapy, three different FDG-PET criteria, European Organization for Research and Treatment of Cancer (EORTC), PET Response Criteria in Solid Tumors (PERCIST), immunotherapy-modified PERCIST (imPERCIST), were compared regarding response evaluation and prognosis prediction using standardized uptake value (SUV) harmonization of results obtained with various PET/CT scanners installed at different centers.

**Materials and methods:**

Malignant melanoma patients (*n* = 27) underwent FDG-PET/CT examinations before and again 3 to 9 months after therapy initiation (nivolumab, *n* = 21; pembrolizumab, *n* = 6) with different PET scanners at five hospitals. EORTC, PERCIST, and imPERCIST criteria were used to evaluate therapeutic response, then concordance of the results was assessed using Cohen’s *κ* coefficient. Log-rank and Cox methods were employed to determine progression-free (PFS) and overall (OS) survival.

**Results:**

Complete metabolic response (CMR)/partial metabolic response (PMR)/stable metabolic disease (SMD)/progressive metabolic disease (PMD) with harmonized EORTC, PERCIST, and imPERCIST was seen in 3/5/4/15, 4/5/3/15, and 4/5/5/13 patients, respectively. Nearly perfect concordance between each pair of criteria was noted (*κ* = 0.939–0.972). Twenty patients showed progression and 14 died from malignant melanoma after a median 19.2 months. Responders (CMR/PMR) showed significantly longer PFS and OS than non-responders (SMD/PMD) (harmonized EORTC: *p* < 0.0001 and *p* = 0.011; harmonized PERCIST: *p* < 0.0001 and *p* = 0.0012; harmonized imPERCIST: *p* < 0.0001 and *p* = 0.0012, respectively).

**Conclusions:**

All harmonized FDG-PET criteria (EORTC, PERCIST, imPERCIST) showed accuracy for response evaluation of ICI therapy and prediction of malignant melanoma patient prognosis. Additional studies to determine their value in larger study populations will be necessary.

## Introduction

Recent breakthrough results from use of immune checkpoint inhibitors (ICIs) have provided a leap forward, which has led to a new era of cancer immunotherapy and cancer treatment paradigm shift [1]. Notably, strategies for inhibiting the anti-programmed death-1 (PD-1)/programmed death-ligand 1 (PD-L1) axis with ICI treatment, including nivolumab and pembrolizumab, have been emerging as novel options for malignant melanoma [2].

Adequate assessment of systemic treatment response is crucial for effective cancer treatment management, which includes effective means to monitor responsiveness of the tumor to systemic therapy, and extremely important for moderation of the high risk of mortality as well as toxic effects known to be associated with available systemic therapeutic regimens. Several recent studies have found the utility of baseline and follow-up ^18^F-fludeoxyglucose (^18^F-FDG) positron emission tomography/computed tomography (PET/CT) results for assessing therapeutic response in cases of malignant melanoma treated with an ICI and also prognosis prediction [3–6]. Criteria commonly used for tumor response shown by PET include assessment of the change in sum of maximum standardized uptake value (SUV_max_) or SUV after correction for lean body mass (SUL_peak_) of up to five lesions, as reported by the European Organization for Research and Treatment of Cancer (EORTC) [7], and shown by the PET Response Criteria in Solid Tumors (PERCIST) [8] and immunotherapy-modified PERCIST (imPERCIST) [6]. However, widespread use of PET for determining treatment response has been limited by differences in the range of SUV among different available PET scanners. To compensate, harmonization among PET models has been used [9, 10]. Another important issue is that until now, treatment response evaluations of patients treated with ICIs have been performed at a single center, while data obtained at multiple centers using various PET scanners have not been utilized. It is considered that more widespread use of PET to determine efficacy could occur should varied PET data obtained at multiple institutions be integrated to better determine treatment response.

This retrospective study sought to evaluate therapeutic response in patients with malignant melanoma treated with ICIs at different medical centers equipped with various PET scanners, and predict prognosis by use of baseline and follow-up ^18^F-FDG PET/CT results with harmonized metabolic markers. Additionally, the utility of three different ^18^F-FDG PET/CT criteria (EORTC, PERCIST, imPERCIST) was examined.

## Materials and methods

### Patients

An appropriate institutional review board at each hospital approved this retrospective multi-center study, including waiving of informed consent requirements. Clinical records were reviewed to identify appropriate patients for analysis. The information systems of five hospitals were screened for cases of malignant melanoma treated with a PD-1 inhibitor or PD-L1 inhibitor therapy from August 2014 to October 2019, and with ^18^F-FDG PET/CT results obtained before and after the start of therapy. Inclusion criteria included (1) ^18^F-FDG PET/CT scanning performed within 3 months before, and from 3 to 9 months after initiation of ICI therapy, and (2) FDG-avid lesions observed in the pretreatment ^18^F-FDG PET/CT examination. History or coexistence of other malignancies, and treatment with other ICIs before the present ICI therapy were used as exclusion criteria.

### Protocol for ^18^F-FDG PET/CT

Eight different whole-body PET/CT scanners were used at the participating institutions; Discovery 600, Discovery 710, Discovery iQ HD and Discovery MI (GE Healthcare, WI, USA), Gemini GXL16, Gemini TF, Ingenuity TF (Philips Medical Systems, Eindhoven, The Netherlands), and Aquiduo (Cannon Medical System, Ohtawara, Japan) (Table 1). Each patient was instructed to fast for at least 4 h prior to the examination. In those with a plasma glucose level < 200 mg/dL, the radiotracer was injected IV at 3–4.5 MBq/kg, followed by 50–70 min of rest before image acquisition. Scans were acquired with an axial field of view from the vertex to mid-thigh or toe. For attenuation correction of the PET emission scan and anatomical orientation, low-dose CT images obtained during PET/CT were used. Reconstruction of the PET/CT images was done with an ordered-subset expectation–maximization algorithm or Bayesian penalized likelihood reconstruction algorithm, as well as with a Gaussian filter using standard reconstruction software supplied by the manufacturer [11, 12]. For optimal harmonization filter calculations, PET data were reconstructed using the default parameters of each institution. An experienced medical physicist had harmonized the acquisition and reconstruction parameters to minimize SUV differences between scanners based on testing with regular phantom studies using region of interest (ROI) and volume of interest (VOI) Analysis Tool (RAVAT) and RC Tool for Harmonization (Nihon Medi-Physics Co., Ltd. Tokyo, Japan) so as to harmonize SUVs obtained with different PET/CT systems in a range advocated by the Japanese Society of Nuclear Medicine using a previously reported method [11, 12].

**Table 1:** Clinical parameters of PET scanners

Scanner	Gemini GXL	Gemini TF	Ingenuity TF	Discovery 600	Discovery 710	Discovery iQ HD	Discovery MI	Aquiduo
Vender	Philips	Philips	Philips	GE	GE	GE	GE	Cannon
PET scanning								
FDG injection dose (MBq/kg)	4	3	3.75	4	3.75	3.75	4	4.5
Scan time (s) for each bed	90	90	90	120	120	180	120	120
TOF	No	Yes	Yes	No	Yes	No	Yes	No
PET reconstruction								
Reconstruction	LOR-RAMLA	3D-OSEM	3D-OSEM	3D-OSEM	3D-OSEM	Q.Clear	Q.Clear	FORE-OSEM
Iterations	2	3	3	2	3	n/a	n/a	4
Subsets	n/a	33	33	16	8	n/a	n/a	14
Penalization factor (*β*)	n/a	n/a	n/a	n/a	n/a	400	700	n/a
Smoothing	n/a	n/a	n/a	Gaussian	Gaussian	n/a	n/a	Gaussian
Matrix	144 × 144	144 × 144	144 × 144	192 × 192	192 × 192	192 × 192	256 × 256	128 × 128
Pixel size (mm)	4 × 4 × 4	4 × 4 × 4	4 × 4 × 4	2.6 × 2.6 × 2.6	3.65 × 3.65 × 3.27	3.13 × 3.13 × 3.26	2.73 × 2.73 × 2.79	3.98 × 3.98 × 2.00
PSF	No	No	No	No	Yes	Yes	Yes	No
FWHM (mm) for harmonization	n/a	5.8	n/a	3.4	8.6	7.1	8.3	n/a
Number of patients	1	1	1	10	7	1	3	3

*FDG* fluorodeoxyglucose, *TOF* time of flight, *LOR-RAMLA* line-of-response row-action maximum likelihood algorithm, *OSEM* ordered-subset expectation maximization, *FORE* fourier rebinning, *PSF* point spread function, *FWHM* full-width at half maximum

### Analysis of images

Local experienced physicians who were board-certified for both diagnostic radiology and nuclear medicine at each institution reviewed the ^18^F-FDG PET/CT images obtained at their hospital in the comparison between the first and second ^18^F-FDG PET/CT scans. An FDG-avid lesion was defined as a focal abnormally increased area of ^18^F-FDG uptake as compared to the background, with or without a corresponding anatomic lesion seen on the CT scan image that was suggestive of metastasis. To obtain the SUV, the VOI was placed manually on a suitable reference fused axial image, defined based on the craniocaudal and mediolateral extent encompassing the entire target lesion, then any avid normal structures were excluded. The freely available software package RAVAT (Nihon Medi-Physics Co., Ltd. Tokyo, Japan) was used to calculate SUV_max_, SUV_mean_, and SUL_peak_.

SUV_max_ was defined as maximum concentration in the target lesion (injected dose/body weight). To determine SUV_peak_, a 1.2-cm diameter volume ROI was placed on the hottest site of the tumor, then normalized (SUV_peak_ × [lean body mass]/[total body mass]) and SUV_mean_ calculations were performed based on the summed SUV in each voxel in the target volume divided by number of voxels within the target volume. Metabolic tumor volume (MTV) was automatically measured inside the tumor VOI with the margin threshold set at 40% of SUV_max_. Then, tumor lesion glycolysis (TLG) was calculated as SUV_mean_ × MTV, with consideration of both metabolic activity and tumor burden. The corresponding values for each lesion in the patient were summed to calculate MTV and TLG.

### Criteria for treatment response

Treatment response was classified as complete metabolic response (CMR), partial metabolic response (PMR), stable metabolic disease (SMD), or progressive metabolic disease (PMD). Based on EORTC, tumor response was also determined, as follows [7]. CMR was defined as complete resolution of ^18^F-FDG uptake within the measurable target lesion making it indistinguishable from the surrounding background with no new ^18^F-FDG-avid lesions. For patients with metabolically active lesions shown in follow-up scanning, the SUV_max_ values of the same lesions (up to a total of five) noted in the baseline and follow-up scans were summed (maximum of two per organ). When the sum of the SUV_max_ values showed a decrease ≥ 25%, tumor response was classified as PMR. PMD indicated a ≥ 25% increase in the sum of the SUV_max_ values or detection of new ^18^F-FDG-avid lesions characteristic of cancer. SMD was used to classify findings other than CMR, PMR, or PMD.

To determine therapeutic response according to PERCIST [8], a 1.2-cm diameter volume ROI was placed on the target lesion and SUL values were calculated. Additionally, the tumor SUL_peak_ value was determined and compared with that of the liver SUL to check if it was 1.5 times or more greater than that of the liver SUL (mean ± 2 standard deviation (SD)) in a 3-cm diameter spherical ROI on the normal right lobe. CMR was the classification when complete resolution of ^18^F-FDG uptake within the target lesion was lower than mean liver activity and indistinguishable from the level of the background blood pool. When metabolically active lesions were noted in a follow-up scan, the SUL_peak_ values of up to five lesions at the baseline and in follow-up examinations were summed (maximum two per organ), and the hottest lesions in each scan selected, thus target lesions noted in follow-up examinations were not necessarily the same as those in baseline images. In cases with an SUL_peak_ sum decreased ≥ 30%, tumor response was classified as PMR. Conversely, when SUL_peak_ sum was increased ≥ 30%, or appearance of new hypermetabolic lesions or ≥ 75% increase in TLG in follow-up ^18^F-FDG PET/CT scan imaging was noted, that was defined as PMD. Cases not defined as CMR, PMR, or PMD received the classification of SMD.

imPERCIST was performed in the same manner as PERCIST, though new lesion appearance alone did not lead to a PMD classification [6], as that was defined only when the increase in the sum of SUL_peak_ values was ≥ 30%. New lesions were included in the SUL_peak_ sum for cases with a higher uptake level than the existing target lesions or when fewer than five target lesions in the baseline scan were detected.

### Statistical analysis

Data are presented as the mean ± SD. Concordance between criteria methods was assessed using Cohen’s *κ* coefficient [13], with level of agreement noted as slight (*κ* < 0.21), fair (*κ* = 0.21–0.40), moderate (*κ* = 0.41–0.60), substantial (*κ* = 0.61–0.80), or nearly perfect (*κ* > 0.80). Progression-free survival (PFS) was defined based on the time elapsed from the start of ICI therapy to date of disease progression revealed in radiological and/or clinical examination results, or death from any cause. Patients with no evidence of progressive disease were censored at the date of the last follow-up examination. Overall survival (OS) was determined as start of ICI therapy until death from any cause. Patients alive at the final follow-up examination were censored, with alive with disease or no evidence of progression used for the classification. Actuarial survival curves were generated using the Kaplan–Meier method, while a log-rank test was employed to examine differences between groups. The SAS software package, version 9.3 (SAS Institute Inc., Cary, NC, USA), was utilized for statistical analyses, with *p* values < 0.05 considered to indicate significance.

## Results

### Patients

Twenty-seven patients [18 males, 9 females; mean (± SD) 67.4 ± 11.3 years old; range 39–86 years] were selected as subjects. For OS calculation, the final follow-up date was April 2020. Baseline ^18^F-FDG PET/CT scanning was performed at a median 27 days (2–90 days) before ICI therapy initiation, while follow-up scanning was done at a median 147 days (90–269 days) following the first ICI administration. ICI initiation and follow-up ^18^F-FDG PET/CT scanning were performed in 4 cycles in 7, 5 cycles in 1, 6 cycles in 3, 7 cycles in 2, 8 cycles in 6, 9 cycles in 3, 11 cycles in 2, and 13 cycles in 3 of the present cases. Patient characteristics are shown in Table 2. The main regimen for nivolumab (*n* = 21) was a dosage of 240 mg every 2 weeks and for pembrolizumab (*n* = 6) was a dosage of 200 mg every 3 weeks, until observation of apparent disease progression or unacceptable toxicity, or treatment discontinuation was decided by the patient or attending physician. Of the 27 enrolled patients, treatment-related adverse events were noted in 3 (11.1%) (rash, interstitial lung disease, diarrhea in 1 each).

**Table 2:** Patient characteristics

Character	*N*	%
Sex		
Male	18	66.7
Female	9	33.3
Age		
Mean	67.4 ± 11.3	
Range	39–86	
Primary site		
Cutaneous	13	48.1
Nasal cavity	4	14.8
Soft tissue	3	11.1
Esophagus	2	7.4
Vulva	2	7.4
Anal	2	7.4
Vagina	1	3.70
Initial stage		
I	3	11.1
II	7	25.9
III	13	48.1
IV	4	14.8
Previous therapy		
Resection	18	66.7
Resection and chemotherapy	7	25.9
Resection, raiotherapy and chemotherapy	2	7.4

### Harmonization effect

In the 27 pretreatment ^18^F-FDG PET/CT examinations, a total of 110 ^18^F-FDG-avid lesions were noted in lymph node (*n* = 40), bone (*n* = 27), soft tissue (*n* = 11), bowel (*n* = 6), lung (*n* = 6), liver (*n* = 5), abdominal cavity or abdominal wall (*n* = 5), nasal cavity (*n* = 4), salivary ground (*n* = 3), vaginal (*n* = 1), vulva (*n* = 1), and anal (*n* = 1) locations. The pretreatment mean SUV_max_ values for the 110 ^18^F-FDG-avid lesions before and after harmonization were 7.31 ± 5.04 (1.84–32.14) and 6.66 ± 4.67 (1.74–29.54), respectively (*p* < 0.0001), while the pretreatment mean SUV_mean_ values before and after harmonization were 4.34 ± 3.22 (1.04–21.87) and 4.06 ± 2.93 (0.98–18.86), respectively (*p* < 0.0001), and the pretreatment mean SUV_peak_ values before and after harmonization were 4.50 ± 3.23 (1.13–21.89) and 4.29 ± 3.07 (1.09–19.80), respectively (*p* < 0.0001). Furthermore, pretreatment mean whole-body MTV values for the 27 cases before and after harmonization were 97.91 ± 260.35 (2.22–1334.57) and 100.09 ± 260.09 (2.22–1334.57), respectively (*p* = 0.99), and the pretreatment mean whole-body TLG values for those cases before and after harmonization were 514.79 ± 1401.74 (4.89–7297.26) and 509.29 ± 1383.53 (5.26–7200.55), respectively (*p* = 0.12).

A total of 179 ^18^F-FDG-avid lesions were observed in bone (*n* = 62), lymph node (*n* = 49), liver (*n* = 17), lung (*n* = 14), soft tissue (*n* = 11), bowel (*n* = 7), abdominal cavity or abdominal wall (*n* = 6), adrenal gland (*n* = 4), nasal cavity (*n* = 2), salivary ground (*n* = 3), pancreas (*n* = 1), vaginal (*n* = 1), vulva (*n* = 1), and anal (*n* = 1) locations in the 27 posttreatment ^18^F-FDG PET/CT examinations. The pretreatment mean SUV_max_ values for those 179 lesions before and after harmonization were 7.52 ± 5.44 (1.47–29.75) and 6.82 ± 4.94 (1.42–28.36), respectively (*p* < 0.0001), while the posttreatment mean SUV_mean_ values before and after harmonization were 4.57 ± 3.67 (0.89–20.96) and 4.27 ± 3.36 (0.88–20.0), respectively (*p* < 0.0001), and the posttreatment mean SUVpeak values before and after harmonization were 4.68 ± 3.65 (0.84–21.92) and 4.43 ± 3.46 (0.82–20.93), respectively (*p* < 0.0001). Furthermore, the posttreatment mean whole-body MTV values for the 27 cases before and after harmonization were 116.69 ± 258.78 (0–1309.15) and 118.95 ± 258.38 (0–1301.21), respectively (*p* = 0.99), and the posttreatment mean whole-body TLG values were 599.64 ± 1121.24 (0–5077.25) and 587.90 ± 1111.02 (0–5054.63), respectively (*p* = 0.047).

### Treatment response assessment

The patient-based mean ΔSUV_max_ value for target lesions based on harmonized EORTC criteria, ΔSUL_peak_ value for target lesions based on harmonized PERCIST, and ΔSUL_peak_ value for target lesions based on harmonized imPERCIST were − 3.52% (− 100% to + 235.3%), − 2.37% (− 100% to + 318.5%), and + 36.8% (− 100% to + 566.6%), respectively.

**Fig. 1:**
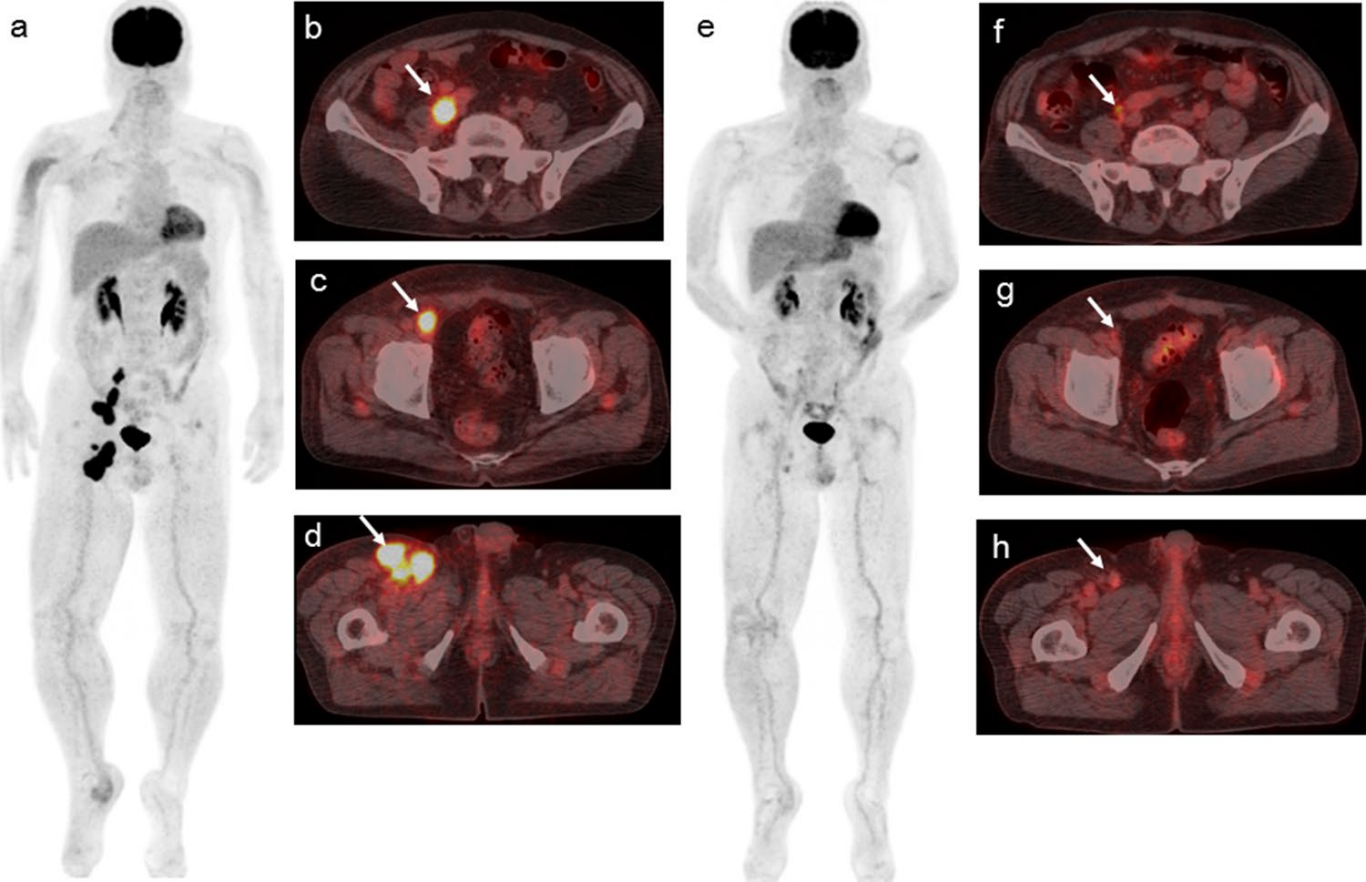
A 66-year-old man with postoperative recurrence malignant melanoma with right pelvic and inguinal nodal metastases received nivolumab. Baseline ^18^F-FDG PET/CT [maximum intensity projection (MIP) (**a**) and fused transaxial (**b**–**d**) images] shows abnormal ^18^F-FDG uptake in the right (**b**) common iliac node metastasis (arrow), **c** external iliac node metastasis (arrow) and **d** inguinal node metastases (arrow). The follow-up ^18^F-FDG PET/CT after nine courses of nivolumab therapy [MIP (**e**) and fused transaxial (**f**–**h**) images] show almost disappearance of ^18^F-FDG uptake in these nodal metastases (arrows). Because post FDG-uptake of these nodal metastases was slightly higher than the surrounding tissue and the reductions of the sum of harmonized SUV_max_ were 83.3% (from 43.95 to 7.33), the status was PMR according to harmonized EORTC criteria. Because post ^18^F-FDG uptake of all three nodal metastases was less than the liver activity, the response status according to harmonized PERCIST and harmonized imPERCIST was CMR. The patient was alive without progression 68.1 months after the initiation of nivolumab. Pretreatment harmonized SUV_max_/SUV_mean_/SUL_peak_ of the right common iliac, external iliac, and inguinal nodal metastases were 14.67/9.32/9.6, 13.09/9.21/9.37, and 16.19/10.09/11.55, respectively. Pretreatment harmonized whole-body MTV and TLG were 72.46 and 714.51, respectively. Posttreatment harmonized SUV_max_/SUV_mean_/SUL_peak_ of the right common iliac, external iliac, and inguinal nodal metastases were 3.26/1.65/1.96, 1.97/1.43/1.62, and 2.13/1.51/1.67, respectively. Posttreatment liver SUL (mean + 2 standard deviations) was 2.33. Posttreatment harmonized whole-body MTV and TLG were 19.35 and 30.58, respectively

**Fig. 2:**
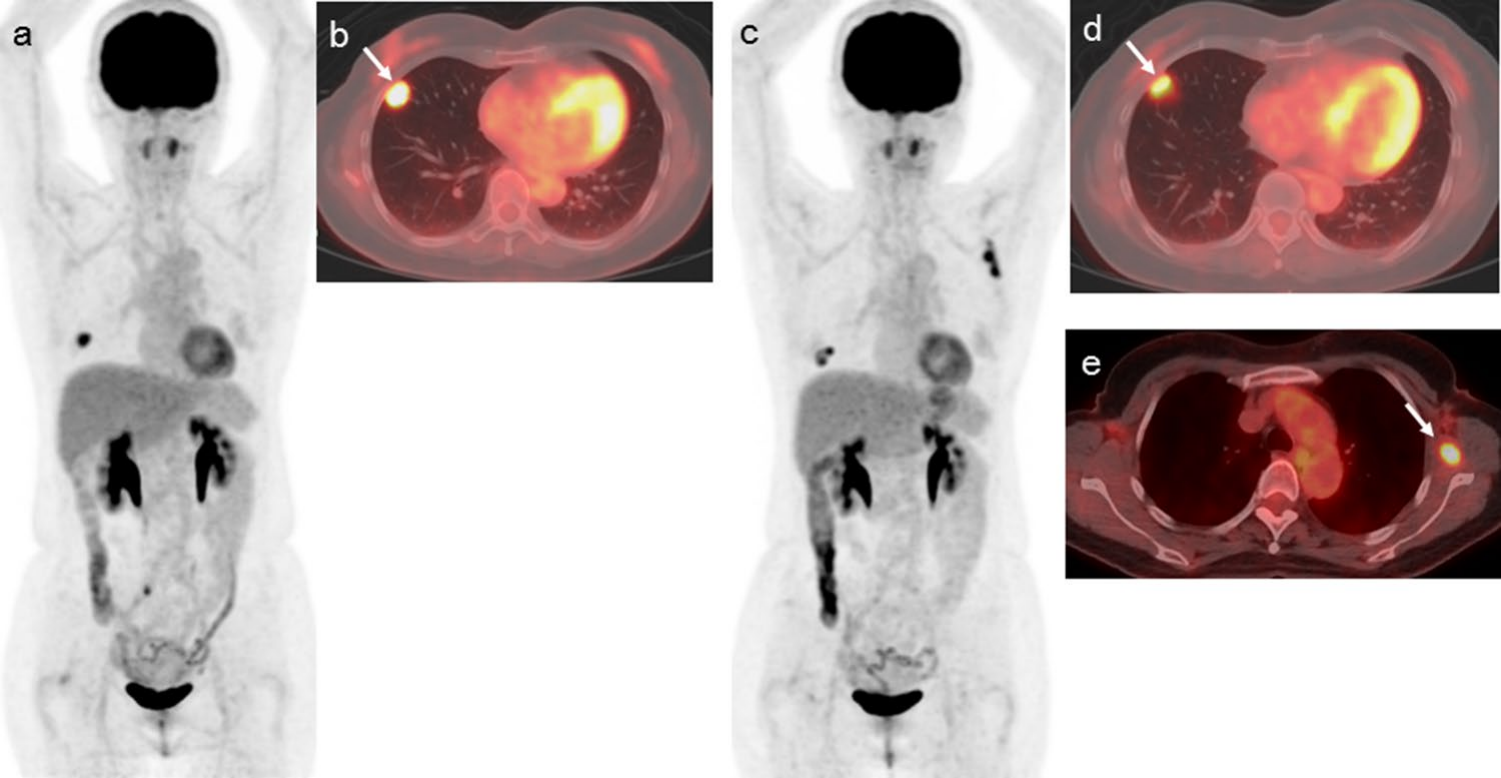
A 58-year-old woman with post-operative and chemotherapeutic recurrence malignant melanoma with lung metastasis received nivolumab. Baseline ^18^F-FDG PET/CT [MIP (**a**) and fused transaxial (**b**) images] shows abnormal ^18^F-FDG uptake in the right lung metastasis (**b**: arrow). The follow-up ^18^F-FDG PET/CT after 13 courses of nivolumab therapy [MIP (**c**) and fused transaxial (**d**, **e**) images] show the slight remission of known lung metastasis (**d**: arrow) with new appearance of subscapular muscle metastasis (**e**: arrow). The status was PMD according to harmonized EORTC criteria and harmonized PERCIST because of new lesions. Because the increase of the sum of the harmonized SUL_peak_ was 44.7% (from 3.87 to 5.6), the status was PMD according to harmonized imPERCIST. The patient exhibited progressive disease at 8.9 months and died 21.9 months after the initiation of nivolumab. Pretreatment harmonized SUV_max_, SUV_mean_, and SUL_peak_ of the right lung metastasis were 6.85, 4.50, and 3.87, respectively. Pretreatment harmonized whole-body MTV and TLG were 2.22 and 10.01, respectively. Posttreatment harmonized SUV_max_/SUV_mean_/SUL_peak_ of the right lung metastasis and left subscapular muscle metastasis were 4.21/3.08/2.44, and 5.53/3.81/3.16, respectively. Posttreatment harmonized whole-body MTV and TLG were 5.6 and 19.16, respectively

Use of harmonized EORTC criteria revealed CMR in 3 (11.1%), PMR in 5 (18.5%), SMD in 4 (14.8%), and PMD in 15 (55.6%) patients, while harmonized PERCIST showed those in 4 (14.8%), 5 (18.5%), 3 (11.1%), and 15 (55.6%), respectively, and harmonized imPERCIST showed those in 4 (14.8%), 5 (18.5%), 5 (18.5%), and 13 (48.1%), respectively. Of the 15 patients classified as PMD based on harmonized EORTC and harmonized PERCIST, appearance of new lesions was noted in 12 and an increase in SUL_peak_ sum for up to 5 lesions ≥ 30% was seen in 3. As for the former 12 patients defined by harmonized EORTC and harmonized PERCIST as PMD due to appearance of new lesions, 10 were classified as PMD and 2 as SMD based on harmonized imPERCIST, due to the definition of that modality. Data for two representative cases are presented in Figs. 1 and 2.

There was concordance noted between harmonized EORTC criteria and harmonized PERCIST response classifications in 25 cases (92.6%), while discordance was seen in 2 (7.4%), with nearly perfect agreement (*κ* = 0.970) for response classification between them (Table 3). As for concordance between harmonized EORTC and harmonized imPERCIST, that was noted in 23 (85.2%) cases, with discordance seen in 4 (14.8%), with nearly perfect agreement (*κ* = 0.939) for response classification between them (Table 4). Furthermore, concordance between harmonized PERCIST and harmonized imPERCIST was seen in 25 (92.6%) cases, and discordance in 2 (7.4%), with nearly perfect agreement (*κ* = 0.972) for response classification between them (Table 5).

**Table 3:** Comparison of treatment response assessments in harmonized EORTC criteria and harmonized PERCIST

	Harmonized EORTC criteria				
	CMR	PMR	SMD	PMD	Total
Harmonized PERCIST					
CMR	3	1	0	0	4
PMR	0	4	1	0	5
SMD	0	0	3	0	3
PMD	0	0	0	15	15
Total	3	5	4	15	27

*EORTC* European Organization for Research and Treatment of Cancer, *PERCIST* positron emission tomography response criteria in solid tumors, *CMR* complete metabolic response, *PMR* partial metabolic response, *SMD* stable metabolic disease, *PMD* progressive metabolic disease

**Table 4:** Comparison of treatment response assessments in harmonized EORTC criteria and harmonized imPERCIST

	Harmonized imPERCIST				
	CMR	PMR	SMD	PMD	Total
Harmonized EORTC critera					
CMR	3	0	0	0	3
PMR	1	4	0	0	5
SMD	0	1	3	0	4
PMD	0	0	2	13	15
Total	4	5	5	13	27

*EORTC* European Organization for Research and Treatment of Cancer, *imPERCIST* immunotherapy-modified positron emission tomography response criteria in solid tumors, *CMR* complete metabolic response, *PMR* partial metabolic response, *SMD* stable metabolic disease, *PMD* progressive metabolic disease

**Table 5:** Comparison of treatment response assessments in harmonized PERCIST and harmonized imPERCIST

	Harmonized imPERCIST				
	CMR	PMR	SMD	PMD	Total
Harmonized PERCIST					
CMR	4	0	0	0	4
PMR	0	5	0	0	5
SMD	0	0	3	0	3
PMD	0	0	2	13	15
Total	4	5	5	13	27

*PERCIST* positron emission tomography response criteria in solid tumors, *imPERCIST* immunotherapy-modified positron emission tomography response criteria in solid tumors, *CMR* complete metabolic response, *PMR* partial metabolic response, *SMD* stable metabolic disease, *PMD* progressive metabolic disease

### Progression-free survival (PFS)

Progressive disease after a median period of 9.9 months (3.4–68.1 months) was noted in 20 (74.1%) of the 27 cases. A comparison of 2-year PFS for responders (CMR/PMR) and non-responders (SMD/PMD) according to harmonized EORTC criteria, harmonized PERCIST, and harmonized imPERCIST showed values of 87.5% vs. 10.5%, 77.8% vs. 11.1%, and 77.8% vs. 11.1%, respectively.

**Fig. 3:**
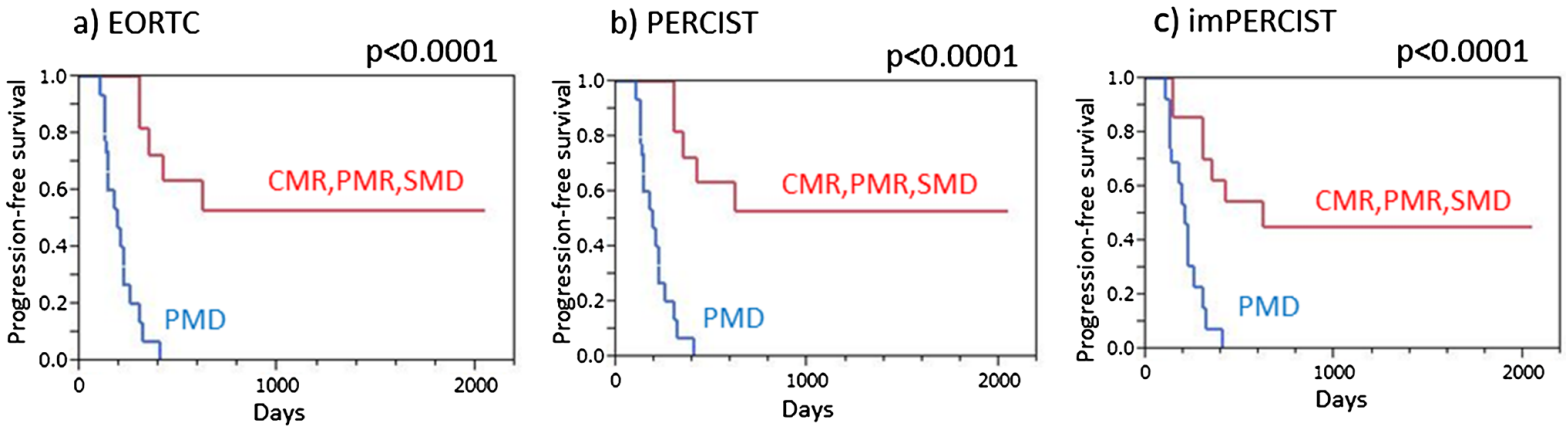
Progression-free survival (PFS) of malignant melanoma patients treated by ICI therapy, with and without progression. **a** EORTC demonstrated that patients with no progression (CMR/PMR/SMD) showed significantly longer PFS than those with PMD (*p* < 0.0001). **b** PERCIST demonstrated that patients with no progression (CMR/PMR/SMD) showed significantly longer PFS than those with PMD (*p* < 0.0001). **c** imPERCIST demonstrated that patients with no progression (CMR/PMR/SMD) showed significantly longer PFS than those with PMD (*p* < 0.0001)

**Fig. 4:**
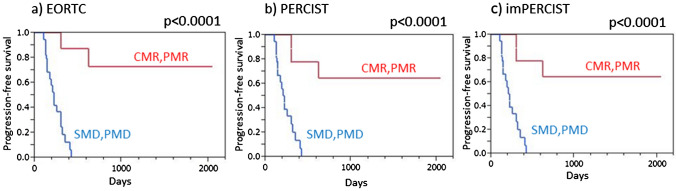
Progression-free survival (PFS) of malignant melanoma patients treated by ICI therapy, with and without response. **a** EORTC demonstrated that responders (CMR/PMR) showed significantly longer PFS than non-responders (SMD/PMD) (*p* < 0.0001). **b** PERCIST demonstrated that responders (CMR/PMR) showed significantly longer PFS than non-responders (SMD/PMD) (*p* < 0.0001). **c** imPERCIST demonstrated that responders (CMR/PMR) showed significantly longer PFS than non-responders (SMD/PMD) (*p* < 0.0001)

Harmonized EORTC, harmonized PERCIST, and harmonized imPERCIST each indicated significantly longer PFS in patients with disease control (CMR/PMR/SMD) than in those with PMD (*p* < 0.0001 for each) (Fig. 3). Similarly, patients classified as responders (CMR/PMR) based on all three criteria showed significantly longer PFS as compared to non-responders (SMD/PMD) (*p* < 0.0001 for each) (Fig. 4).

### Overall survival (OS)

Of the 27 patients, 14 (51.9%) died from a malignant melanoma after a median 19.2 months (4.6–68.1 months). A comparison of 2-year OS for responders (CMR/PMR) and non-responders (SMD/PMD) according to harmonized EORTC, harmonized PERCIST, and harmonized imPERCIST revealed values of 62.5% vs. 15.8%, 66.7% vs. 11.1%, and 66.7% vs. 11.1%, respectively.

**Fig. 5:**
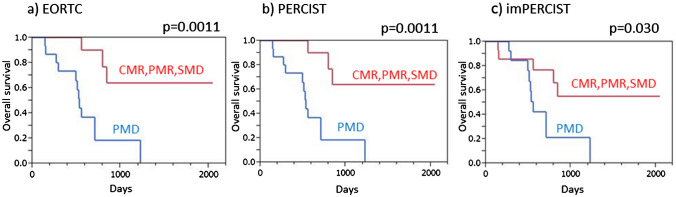
Overall survival (OS) of malignant melanoma patients treated by ICI therapy, with and without progression. **a** EORTC demonstrated that patients with no progression (CMR/PMR/SMD) showed significantly longer OS than those with PMD (*p* = 0.0011). **b** PERCIST demonstrated that patients with no progression (CMR/PMR/SMD) showed significantly longer OS than those with PMD (*p* = 0.00011). **c** imPERCIST demonstrated that patients with no progression (CMR/PMR/SMD) showed significantly longer OS than those with PMD (*p* = 0.030)

**Fig. 6:**
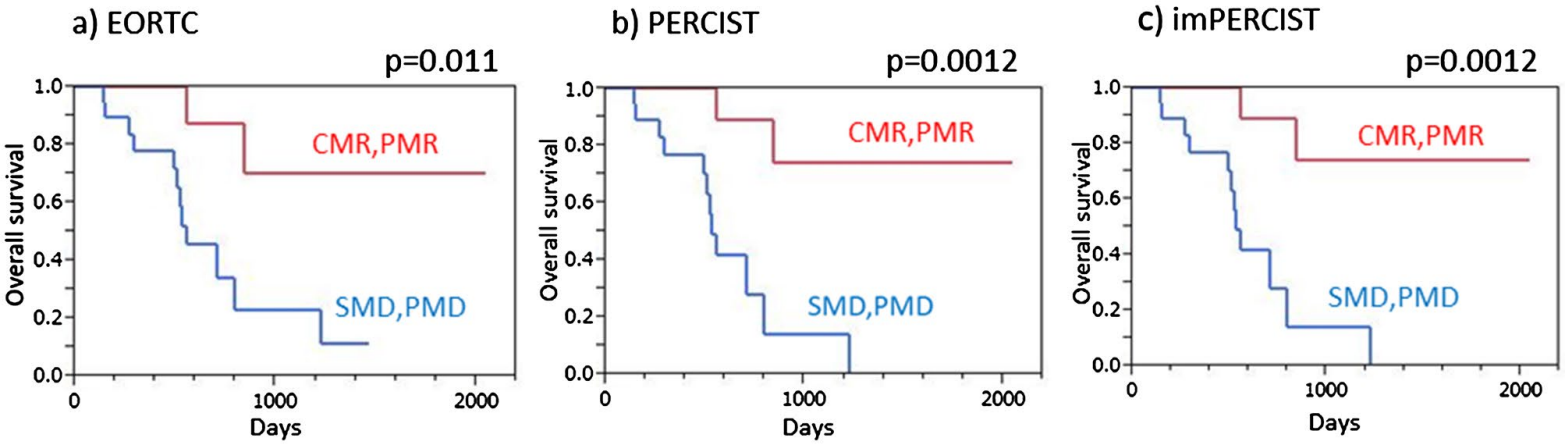
Overall survival (OS) of malignant melanoma patients treated by ICI therapy, with and without response. **a** EORTC demonstrated that responders (CMR/PMR) showed significantly longer OS than non-responders (SMD/PMD) (*p* = 0.011). **b** PERCIST demonstrated that responders (CMR/PMR) showed significantly longer OS than non-responders (SMD/PMD) (*p* = 0.0012). **c** imPERCIST demonstrated that responders (CMR/PMR) showed significantly longer OS than non-responders (SMD/PMD) (*p* = 0.0012)

Harmonized EORTC, harmonized PERCIST, and harmonized imPERCIST each indicated significantly longer OS in patients with disease control (CMR/PMR/SMD) than in those with PMD (harmonized EORTC: *p* = 0.0011, harmonized PERCIST: *p* = 0.00011, harmonized imPERCIST: *p* = 0.030) (Fig. 5). Similarly, patients classified as responders (CMR/PMR) according to all three criteria showed significantly longer OS as compared to non-responders (SMD/PMD) (harmonized EORTC: *p* = 0.011, harmonized PERCIST: *p* = 0.0012, harmonized imPERCIST: *p* = 0.0012) (Fig. 6).

## Discussion

The present is the first known study conducted to evaluate therapeutic response of patients with a malignant melanoma who were treated with ICIs at multiple medical institutions equipped with a variety PET scanners, with prognosis based on baseline and follow-up ^18^F-FDG PET/CT results using harmonized metabolic markers also assessed. The findings clarified that ^18^F-FDG PET/CT results obtained before and again from 3 to 9 months after initiation of ICI therapy using harmonized metabolic markers from eight types of PET scanners in place at five different hospitals were useful to evaluate tumor response as well as prognosis prediction in malignant melanoma patients who received ICI therapy. The impact of this study is considered to be high for clinical practice settings as well as multicenter trials. Use of different types of PET/CT scanners at the same institution is becoming common, thus methods for harmonization of PET quantitative values are needed in both clinical settings and for trials conducted in cooperation among multiple centers. Previously established harmonization programs such as the EANM/EARL program [9] and Quantitative Imaging Biomarker Alliance (QIBA/UPICT) [10] have provided useful comparisons of SUV metrics among different systems.

Comparisons of PERCIST and imPERCIST for evaluating response to ICI treatment in malignant melanoma patients, and prediction of OS were presented in an interesting study by Ito et al. [6], though there are no known reports of comparisons of EORTC criteria (SUV_max_), PERCIST, and imPERCIST (SUL_peak_). In their investigation, Ito et al. found that imPERCIST was superior for OS, while all three harmonized ^18^F-FDG PET criteria showed very high concordance of CMR/PMR/SMD/PMD in the present study, as well as accuracy regarding evaluation of response to ICI therapy and prediction of prognosis in malignant melanoma patients. A potential reason for this difference may have been the patient population, along with definitions of early (2–4 cycles of ICI) in their series and late (4–13 cycles of ICI, median 8 cycles) response for the assessments in our series.

Immune cell infiltration can delay tumor shrinkage or even cause a temporary size increase (pseudoprogression), thus assessment of tumor response following ICI treatment can be difficult. Several different criteria for use with ^18^F-FDG PET/CT findings have been proposed to determine response to that treatment, such as PET/CT criteria for early prediction of response to immune checkpoint inhibitor therapy (PECRIT) [3], PET response evaluation criteria for immunotherapy (PERCIMT) [4], imPERCIST [6], and immune PERCIST (iPERCIST) [5], though an optimal evaluation method has yet to be established. The present criteria were established for early prediction following the start of ICI treatment (2 ~ 4 cycles). While pseudoprogression must be considered in the early phase following treatment initiation, that was not observed in any of our patients, which might have been due to the late (≥ 4 cycles) response assessment.

Several studies have presented results demonstrating the usefulness of ^18^F-FDG PET/CT for assessing ICI therapeutic response, especially early response (2 ~ 4 cycles) [3–6]. Cho et al. [3] showed analysis of PECRIT, which includes change in lesion size combined with change in FDG avidity shown by ^18^F-FDG PET/CT, in 20 advanced melanoma patients after 1 cycle of ICI monotherapy (ipilimumab, nivolumab, or BMS-936559). Criteria that included SD shown by RECIST 1.1 and an SUL_peak_ increase > 15.5% in the hottest lesion shown by ^18^F-FDG PET/CT were found to be accurate for predicting treatment response after 4 months, and they reported values for sensitivity, specificity, and accuracy of 100%, 93%, and 95%, respectively. In another study, Anwar et al. [4] evaluated 41 metastatic melanoma cases after 4 cycles of ipilimumab PERCIMT using absolute number of new lesions rather than metabolic parameter changes (i.e., SUV) shown by ^18^F-FDG PET/CT and reported that those criteria, including evidence of four or more new lesions < 1 cm in functional diameter, were accurate for predicting clinical benefit prediction, with sensitivity and specificity of 84% and 100%, respectively. As noted earlier, Ito et al. [6] were the first to present imPERCIST, in which new lesion appearance is not used to define PMD. They analyzed 60 metastatic melanoma patients and noted that a ≥ 30% increase in SUL_peak_ sum in up to 5 measured lesions shown by ^18^F-FDG PET/CT accurately reflected PMD after 2–4 cycles of ipilimumab. Using iPERCIST, two new categories for response to PMD were introduced by Goldfarb et al. [5], unconfirmed (UPMD) and confirmed (CPMD). Analyses of the results of 28 non-small cell lung cancer patients receiving nivolumab indicated that evidence of metabolic progression observed at 8 weeks (after 4 cycles) should be confirmed by another ^18^F-FDG PET/CT examination 4 weeks later, while the usefulness of iPERCIST for differentiation of responders from non-responders and OS prediction was also noted (*p* = 0.0003).

This study has some limitations. Since the results were obtained from a retrospective review of a small selected patient group, selection bias may have had an influence, as PET/CT imaging was used at the discretion of the referring physician. A prospective study with a much larger population is needed. Furthermore, the time period between start of ICI therapy and follow-up imaging was not standardized, which might have had effects related to changes in tumor FDG uptake and number of lesions detected. On the other hand, the present results reflect typical usage of ^18^F-FDG PET/CT in clinical settings, and a clear correlation between PET response criteria and PFS or OS was shown, suggesting that response assessment by PET is acceptable for use in clinical practice.

In conclusion, the three harmonized ^18^F-FDG PET criteria (EORTC, PERCIST, imPERCIST) used in the present study demonstrated high concordance for CMR/PMR/SMD/PMD, as well as accuracy for evaluation of response to ICI therapy and prediction of prognosis in cases of malignant melanoma. Nevertheless, future studies will be needed with larger study populations to better determine the value of these methods.
